# Diabetes mellitus and its association with tuberculosis clinical presentation and treatment outcomes: results from a prospective cohort study in Ghana

**DOI:** 10.3389/fpubh.2026.1755479

**Published:** 2026-04-09

**Authors:** Monica Baaba Jones, Kwadwo Ansah Koram, Francis Anto, Michael Lauzardo, Blanca I. Restrepo, Margaret Lartey, Hafisatu Gbadamosi, Awewura Kwara

**Affiliations:** 1 Department of Epidemiology and Disease Control, School of Public Health, College of Health Sciences, University of Ghana, Accra, Ghana; 2 Noguchi Memorial Institute for Medical Research, College of Health Sciences, University of Ghana, Accra, Ghana; 3 Department of Medicine, Division of Infectious Diseases and Global Medicine, Emerging Pathogens Institute, University of Florida, Gainesville, FL, United States; 4 School of Public Health, University of Texas Health Science Centre at Houston, Brownsville, TX, United States; 5 South Texas Diabetes and Obesity Institute, University of Texas Rio Grande Valley, Edinburg, TX, United States; 6 Department of Medicine & Therapeutics, University of Ghana Medical School, College of Health Sciences, University of Ghana, Accra, Ghana; 7 Department of Radiology, Korle Bu Teaching Hospital, Accra, Ghana

**Keywords:** diabetes mellitus, TB clinical presentation, TB treatment outcomes, TB-diabetes comorbidity, tuberculosis

## Abstract

**Background:**

Tuberculosis (TB)-diabetes is a growing public health threat in TB-endemic settings. We aimed to determine diabetes prevalence among TB patients in Greater Accra and its association with TB clinical presentation and treatment outcomes.

**Methods:**

We enrolled 204 adults (≥20 years) with bacteriologically confirmed pulmonary TB across 14 health facilities. At treatment initiation, participants were screened for diabetes using self-report, fasting plasma glucose, and glycated hemoglobin, following standard diagnostic criteria. Baseline characteristics were recorded, and participants were followed to determine TB treatment outcomes. Associations between diabetes, baseline characteristics and treatment outcomes were assessed using bivariate and multivariate analysis in STATA. Adjusted odds ratios (AORs) and risk ratios (RRs) were estimated with 95% confidence intervals (CIs).

**Results:**

The median age of participants was 40.5 years (IQR 30.5–50.5), and 72.5% were male. The prevalence of baseline diabetes was 22.1% (45/204), including 30 newly diagnosed and 15 previously diagnosed participants on treatment. Among 166 chest X-rays, cavities were less frequent in TB-diabetes than TB-only participants (51.2% vs. 72.5%, *P* = 0.014). Diabetes was associated with age ≥60 years (AOR 5.7, 95% CI 1.7–19.3), body mass index ≥25 kg/m^2^ (AOR 5.4, 95% CI 1.4–21.9), and family history of diabetes (AOR 3.7, 95% CI 1.5–9.4). Overall, 90.7% had favorable TB treatment outcomes, with unfavorable outcomes in 6.8% of TB-diabetes and 10.1% of TB-only participants (RR 0.68, 95% CI 0.2–2.2).

**Conclusion:**

Diabetes was common among TB patients, with many previously undiagnosed. Despite similar treatment outcomes to TB-only participants, routine diabetes screening is recommended for early detection and management.

## 1 Introduction

The convergence of tuberculosis (TB) and diabetes mellitus presents a growing challenge to TB control and patient outcomes, especially in low- and middle-income countries (LMICs). TB remains a leading global cause of death, with over 10 million new cases and more than 1 million deaths each year (1). Diabetes, a known TB risk factor (2), impairs immune response and is associated with unfavorable TB treatment outcomes (3, 4). Population attributable risk estimates from a systematic review and meta-analysis of observational studies suggest that diabetes may contribute to up to 25% of TB-related mortality (5). Globally, the prevalence of diabetes is rising rapidly, currently affecting approximately 830 million people, many of whom reside in LMICs (6). In Africa, where TB is endemic, diabetes cases are projected to increase from 16 million recorded in 2017 to 41 million by 2045 (7), heightening concerns in high-TB burden settings such as Ghana.

Global estimates show wide variations in diabetes prevalence among TB patients (8). Estimates from sub-Saharan Africa are similarly inconsistent and highly variable (9). In Ghana, diabetes affects about 6.5% of adults (10). Although bi-directional TB–diabetes screening has been instituted (11), routine diabetes screening among TB patients is limited. In addition, in-country data on the epidemiological and clinical profile of TB patients with diabetes remain sparse. A large cross-sectional study conducted in a tertiary facility found a higher prevalence of diabetes among TB patients than in the general population, as well as high proportions of treatment failure among patients with diabetes (12).

Our study adds to this body of evidence by utilizing a multifacility, prospective design to provide further insights into TB-diabetes comorbidity in Ghana. Understanding the prevalence and impact of diabetes among TB patients is critical to guide local screening and management strategies, and to contribute to the regional evidence on TB-diabetes comorbidity. We determined the prevalence of diabetes among TB patients and its relationship with TB clinical presentation and treatment outcomes in the Greater Accra Region.

## 2 Materials and methods

### 2.1 Study design and population

We conducted a prospective cohort study among adult outpatients (≥20 years) with Xpert- or smear-positive pulmonary TB initiating standard 6-month first-line treatment across 14 health facilities. At treatment initiation, consenting patients were screened for diabetes and assessed for TB presentation, comorbidities, and key sociodemographic, anthropometric, and behavioral characteristics. TB diagnosis was made using the Xpert MTB/RIF assay (Cepheid, USA). Participants were assessed monthly for clinical outcomes until end-of-TB treatment outcomes were determined or they exited the study. The median follow-up was 6 months (range 1–8 months). Patients with drug-resistant TB, pregnancy, or modified treatment regimens were excluded.

### 2.2 Study setting

Greater Accra Region (GAR), Ghana's most urbanized region with a population of 5.4 million in 2021 (13), has high TB notification rates (14) and longstanding non-communicable disease burden (15). Fourteen clinics were selected based on: (1) district TB notification ≥30/100,000 with ≥70 TB cases in 2018, (2) ≥3 bacteriologically confirmed PTB cases from January–June 2019, and (3) facility head approval. These facilities managed nearly 90% of TB cases in GAR in 2018.

### 2.3 Sample size and recruitment

Sample size estimation addressed two primary study objectives. First, to estimate the baseline prevalence of diabetes among TB patients, the sample size was calculated using the single proportion formula *n* = (*z*^2^ × *p* × (1 − *p*))/*d*^2^ (16), with *z* = 1.96, *p* = 15.1% [previous estimate (17)], and *d* = 5%, yielding 197. Second, a separate sample size for comparing end-of-TB treatment outcomes by baseline diabetes status was calculated. Using data from the previous Ghana study (12) and standard methods for comparing two proportions (18), this yielded a minimum of 112. The larger estimate of 197 was therefore selected to ensure adequate power for both objectives. After finite population correction (*N* = 1,476) (19) and adjusting for an anticipated 15% non-response rate (20), the final target sample size was 204 participants. Recruitment occurred consecutively between 1 October 2019 and 7 July 2020.

### 2.4 Data collection

#### 2.4.1 Diabetes screening at baseline

At enrolment, participants reported any prior diabetes diagnosis and anti-diabetic medication use. Nurses counseled participants to fast for at least 8 h and return the next morning for venous blood draw. Fasting plasma glucose (FPG) was assessed using the enzymatic UV (hexokinase) method on a DxC 700 AU clinical chemistry analyser (Beckman Coulter, CA, USA). HbA1c was measured by turbidimetric inhibition immunoassay on a Cobas Integra 400 plus analyser (Roche Diagnostics, NY, USA). Samples were processed at MDS-Lancet Laboratories, Ghana (ISO 15189:2012).

Participants were classified at baseline as TB-diabetes (TB patients with diabetes) or TB-only (TB patients without diabetes). Diabetes was defined as HbA1c ≥6.5%, FPG ≥7.0 mmol/L, or a prior diagnosis. It was further classified as newly diagnosed if identified during the study, and previously diagnosed if participants reported a prior diagnosis. For six participants, one of the two diabetes screening tests was missing; hence, classification was based on the available result. Participants were classified by baseline diabetes status for all analyses, regardless of any change during follow-up.

#### 2.4.2 Baseline characteristics of study participants

Questionnaire data covered age, education, socio-economic status [based on living standards (21)], smoking, and alcohol use. Smoking status was classified as never, former, or current. Alcohol use was categorized as abstainer, moderate (< 3 drinks/day or < 6 drinks/occasion), or heavy (≥3 drinks/day or ≥6 drinks/occasion), based on the first three AUDIT questions (22). Weight (kg), height (m), and blood pressure (mmHg) were measured. BMI was calculated as kg/m^2^, anemia was defined as hemoglobin < 12 mg/dL (for females) or < 13 mg/dL (for males) (23), and hypertension as ≥140/90 mmHg or a self-reported history. HIV status and antiretroviral therapy (ART) use were abstracted from records.

#### 2.4.3 TB clinical presentation

Previous TB history and baseline sputum results (bacillary load) were obtained from records. Sputum bacillary load was classified based on Xpert results as very low, low, medium, or high. For five participants diagnosed based on smear microscopy only, results were mapped to Xpert categories as follows: “scanty” to very low, “1+” to low, “2+” to medium, and “3+” to high. TB symptoms were self-reported.

Posterior-anterior chest radiographs were obtained before or at the start of TB treatment. A radiologist, blinded to participants' diabetes status, reviewed the images for cavitary lesions and pulmonary infiltrates. Cavities were defined as abnormal air-filled spaces, typically with a wall, within the lung parenchyma. Pulmonary infiltrates were defined as abnormally increased density of the lung parenchyma. The number of lungs affected by infiltrates was recorded as unilateral (one lung) or bilateral (both lungs). Lung zones involved and the presence of lower lung field TB (middle and/or lower zones) were noted. Images that were initially considered inconclusive were reviewed by a second radiologist, after which a single final classification was agreed upon through discussion. Radiographic features were classified as indeterminate when their presence or absence could not be reliably assessed due to poor image quality or diagnostic uncertainty.

#### 2.4.4 Follow-up assessments and TB treatment outcomes

Diabetes screening was repeated at approximately 3-month intervals after baseline. Glucose control among participants with previously diagnosed diabetes was assessed using HbA1c and classified as Good (<7.0%), Fair (7%−9%), or Poor (>9.0%) (24). Participants were asked about anti-diabetic medication use.

TB treatment adherence was assessed monthly and calculated as the proportion of doses taken (i.e., unmissed doses ÷ 168 doses) (25). Adherence was classified as “Good” (100%), “Fair” (90%−99%), or “Poor” (<90%). Follow-up sputum smear results and information on treatment completion, interruptions, discontinuation or death were extracted from medical records.

End-of-TB treatment outcomes were classified according to WHO guidelines (26). Favorable outcomes included cure (smear-negative at the end of treatment and on at least one previous occasion) or treatment completed (completed treatment without evidence of failure but without available negative smear results at the end of treatment). Unfavorable outcomes included treatment failure (smear-positive at month 5 or later), death (during TB treatment), or lost to follow-up (LTFU; treatment interrupted for ≥2 consecutive months).

### 2.5 Statistical analysis

A total of 215 participants were recruited. Eleven lacked baseline diabetes screening and were excluded from baseline analyses, leaving 204 participants. Missing data were handled as follows: descriptive statistics used the total analytic sample as the denominator, with missing data reported for each variable, and multivariable analyses included only complete cases. Chest radiographs were available for 166 participants, and analyses were restricted to this subgroup. Indeterminate radiologic features were excluded from the primary analysis. However, in sensitivity analysis, these observations were included as a separate category to assess whether their exclusion influenced the results. The number of indeterminate observations was seven for cavitary lesions, two for pulmonary infiltrates, and two for lower lung field TB.

End-of-TB treatment outcome analyses included 193 participants −10 who transferred out and one who discontinued due to adverse events were excluded. Sputum results were unavailable for those who could not produce sputum, those who died, or were LTFU −19 participants lacked smear results at Month 2, 73 at Month 5, and 66 at the end of treatment. Outcomes were classified according to WHO guidelines, regardless of missing sputum results.

Analyses were conducted using Stata 15.0 (StataCorp, TX, USA). Continuous variables were assessed for normality using skewness and kurtosis statistics, supplemented by histogram inspection. They were summarized as medians with interquartile ranges. Socioeconomic status was derived using a principal component analysis and grouped into low, middle, and high tertiles. Age was categorized as 20–39, 40–59, and ≥60 years. Baseline diabetes prevalence was calculated as the proportion of enrolled participants with newly or previously diagnosed diabetes at the start of TB treatment.

Baseline characteristics of TB-diabetes and TB-only participants were compared using chi-square, Fisher's exact, and Wilcoxon rank-sum tests, as appropriate. Variables with *P* < 0.20 in univariate logistic regression and biologically plausible factors were included in a multivariable logistic regression model adjusting for age, sex, education, alcohol use, BMI, and family history of diabetes. Crude and adjusted odds ratios (ORs) with 95% confidence intervals (CIs) were computed. A screening threshold of 0.20 was used because a significance level of 0.05 may fail to identify important confounders during initial screening, and this approach has been used in other studies (27). Age was analyzed both as a categorical and continuous variable to estimate the change in odds of diabetes associated with each 1-year increase. Sex was recorded as male or female at birth, based on patient records.

TB presentation (symptoms, radiographic features, and sputum bacillary load) was compared between TB-diabetes and TB-only participants using chi-square or Wilcoxon rank-sum tests, as appropriate. Month-2 smear positivity was summarized as the proportion remaining smear-positive at the end of the intensive phase. Differences were assessed using chi-square or Fisher's exact tests, as appropriate.

End-of-TB treatment outcomes were compared between TB-diabetes and TB-only groups using risk ratios (RRs). Crude RRs with 95% confidence intervals (CIs) were calculated separately for favorable and unfavorable outcomes to compare proportions. Adjusted RRs were not estimated due to a lack of association between diabetes and treatment outcomes in the primary analysis.

Statistical significance was set at *P* < 0.05 (two-tailed).

### 2.6 Ethical considerations

Ethical approval was obtained from the Ghana Health Service Ethics Review Committee (GHS-ERC 004/05/19) and the 37 Military Hospital Institutional Review Board (37MH-IRB/PhD/IPN/322/2019). Permission to conduct the study was granted by the National Tuberculosis Programme, and regional and district health authorities. Written informed consent was obtained from all participants.

## 3 Results

### 3.1 Characteristics of study participants

A total of 204 participants aged 20–82 years (median 40.5; IQR 30.0–50.5) were included in the baseline analysis. Most were male (72.5%) and had some formal education (88.7%; 1 missing).

Median BMI was 19.0 kg/m^2^ (IQR 17.2–21.3; 2 missing). Co-morbidities included HIV (14.2%; 1 missing), hypertension (16.2%; 15 missing), and anemia (76.0%; 9 missing). Most participants (95.1%) were new TB cases. A family history of diabetes was reported by 14.2%.

Current smoking and heavy alcohol use were reported by 13.8% (1 missing) and 35.3% of participants, respectively (Table 1).

**Table 1:** Baseline characteristics of study participants.

Variables	Cohort (*N* = 204)	TB-diabetes (*N* = 45)	TB-only (*N* = 159)	*P* value
	*n* (col%)	*n* (row%)	*n* (row%)	
Age group, years				0.006
20–39	95 (46.6)	15 (15.8)	80 (84.2)	
40–59	91 (44.6)	21(23.1)	70 (76.9)	
≥60	18 (8.8)	9 (50.0)	9 (50.0)	
Median age (IQR)	40.5 (30.0, 50.5)	48.0 (36.0,57.0)	39.0 (28.0, 48.0)	
Sex				0.168
Male	148 (72.5)	29 (19.6)	119 (80.4)	
Female	56 (27.5)	16 (28.6)	40 (71.4)	
Formal education				0.070
None	23 (11.3)	8 (34.8)	15 (65.2)	
Primary	26 (12.7)	2 (7.7)	24 (92.3)	
Secondary or higher	155 (76.0)	35 (22.6)	120 (77.4)	
Socio-economic status				0.630
Low	70 (34.5)	13 (18.6)	57 (81.4)	
Middle	70 (34.5)	16 (22.9)	54 (77.1)	
High	63 (31.0)	16 (25.4)	47 (74.6)	
BMI, kg/m^2^				0.003
< 18.5	86 (42.1)	14 (16.3)	72 (83.7)	
18.5–24.9	102 (50.0)	23 (22.5)	79 (77.5)	
≥25.0	14 (6.9)	8 (57.1)	6 (42.9)	
HIV status				0.836
Negative	174 (85.3)	39 (22.4)	135 (77.6)	
Positive	29 (14.2)	6 (20.7)	23 (79.3)	
Hypertension				0.858
Non-hypertensive	156 (76.5)	34 (21.8)	122 (78.2)	
Hypertensive	33 (16.2)	9 (27.3)	24 (72.7)	
Anemia				
Non-anemic	40 (19.6)	8 (20.0)	32 (80.0)	
Anemic	155 (76.0)	33 (21.3)	122 (78.7)	
TB treatment history				0.462
New	194 (95.1)	42 (21.6)	152 (78.4)	
Previously treated	10 (4.9)	3 (30.0)	7 (70.0)	
Family history of diabetes				< 0.001
No	146 (71.6)	30 (20.5)	116 (79.5)	
Yes	29 (14.2)	14 (48.3)	15 (51.7)	
Unknown	29 (14.2)	1 (3.4)	28 (96.6)	
Smoking status				0.574
Never smoker	142 (69.9)	33 (23.2)	109 (76.8)	
Former smoker	33 (16.3)	7 (21.2)	26 (78.8)	
Current smoker	28 (13.8)	4 (14.3)	24 (85.7)	
Alcohol use				0.160
Abstainer	88 (43.1)	19 (21.6)	69 (78.4)	
Moderate drinker	44 (21.6)	14 (31.8)	30 (68.2)	
Heavy drinker	72 (35.3)	12 (16.7)	60 (83.3)	

IQR, interquartile range; BMI, body mass index, HIV, human immunodeficiency virus; TB, tuberculosis.

### 3.2 Diabetes prevalence and its determinants among study participants

At baseline, 22.1% (45/204; 95% CI 16.5–28.3) had diabetes—two-thirds (30) were newly diagnosed. The remaining 15 had been diagnosed 1–5 years prior to the study—four were on insulin, 10 on metformin, and one on other hypoglycaemic agents. More than half (8/15) of previously diagnosed participants had HbA1c > 9%. Newly diagnosed participants were younger than those previously diagnosed (median age 40.5 vs. 57.0 years; *P* = 0.003).

In the multivariable logistic regression analysis, which accounted for sex, education, alcohol use, BMI, and family history of diabetes, factors independently associated with baseline diabetes were age ≥60 years (COR 5.33, 95% CI 1.82–15.64; *P* = 0.002; AOR 5.69, 95% CI 1.67–19.31; *P* = 0.005), BMI ≥25 kg/m^2^ (COR 4.58, 95% CI 1.44–14.55; *P* = 0.010; AOR 5.43, 95% CI 1.35–21.87; *P* = 0.017), and a family history of diabetes (COR 3.61, 95% CI 1.57–8.27; *P* = 0.002; AOR 3.71, 95% CI 1.47–9.40; *P* = 0.006) (Table 2). Further analysis treating age as a continuous variable showed that each one-year increase raised the odds of diabetes by approximately 5% (COR = 1.05, 95% CI 1.02–1.08, *P* < 0.001; AOR = 1.05, 95% CI 1.01–1.09, *P* = 0.009).

**Table 2:** Participant characteristics associated with diabetes at the start of TB treatment.

Variables	Cohort(N)	OR [95% CI]	*P*-value	AOR [95% CI]	*P*-value
Age group, years	204				
20–39	95	Reference		Reference	
40–59	91	1.60 [0.77–3.34]	0.211	1.32 [0.58–3.03]	0.508
≥ 60	18	5.33 [1.82–15.64]	0.002	5.69 [1.67–19.31]	0.005
Sex	204				
Male	148	Reference		Reference	
Female	56	1.64 [0.81–3.33]	0.170	0.81 [0.31–2.09]	0.663
Formal education	204				
None	23	Reference		Reference	
Primary	26	0.16 [0.03–0.84]	0.030	0.17 [0.03–1.04]	0.055
Secondary or higher	155	0.54 [0.21–1.40]	0.207	0.45 [0.15–1.35]	0.155
Alcohol use	204				
Abstainer	88	Reference		Reference	
Moderate drinker	44	1.69 [0.75–3.82]	0.203	1.71 [ 0.66–4.43]	0.269
Heavy drinker	72	0.73 [0.33–1.62]	0.434	1.04 [ 0.40–2.71]	0.943
BMI, kg/m^2^	202				
< 18.5	86	0.67 [0.32–1.40]	0.283	0.85 [0.37–1.95]	0.696
18.5–24.9	102	Reference			
≥25.0	14	4.58 [1.44–14.55]	0.010	5.43 [1.35–21.87]	0.017
Family history of diabetes^a^	204				
No	146	Reference		Reference	
Yes	29	3.61 [1.57–8.29]	0.002	3.71 [1.47–9.40]	0.006

CI, confidence interval; OR, crude odds ratio; AOR, adjusted odds ratio; BMI, body mass index.

^a^Includes 29 participants unsure of their family history of diabetes; results not shown in the table.

### 3.3 TB clinical presentations by diabetes status

Almost all participants (199/204; 97.5%) were diagnosed using Xpert MTB/RIF. About 43% (87/204) of participants presented with high mycobacterial load. Bacillary load did not differ significantly between TB-diabetes and TB-only participants (*P* = 0.419) (Table 3).

**Table 3:** TB clinical presentation of study participants at the start of TB treatment.

Variables	Cohort	TB-diabetes	TB-only	*P* value
	*n* (col%)	*n* (col%)	*n* (col%)	
Sputum bacillary load	*N* = 204	*N* = 45	*N* = 159	0.419
Very low	13 (6.4)	2 (4.4)	11 (6.9)	
Low	44 (21.6)	13 (28.8)	31 (19.5)	
Medium	60 (29.4)	10 (22.2)	50 (31.5)	
High	87 (42.6)	20 (44.4)	67 (42.1)	
Reported TB symptoms	*N* = 204	*N* = 45	*N* = 159	
Cough	203 (99.5)	45 (100.0)	158 (99.4)	1.000
Sputum production	191 (93.6)	42 (93.3)	149 (94.3)	0.731
Haemoptysis	42 (20.6)	10 (22.2)	32 (20.1)	0.759
Shortness of breath	132 (64.7)	29 (66.40)	103 (65.2)	0.926
Chest pain	154 (75.5)	34 (75.6)	120 (75.5)	0.991
Weight loss	189 (92.6)	39 (86.7)	150 (94.3)	0.104
Night sweats	129 (63.2)	28 (62.20)	101 (63.2)	0.873
Radiological presentation	*N* = 166	*N* = 39	*N* = 127	
Cavitary lesions	107 (64.5)	20 (51.2)	87 (72.5)	0.014
Pulmonary infiltrates	146 (88.0)	31 (79.5)	115 (92.0)	0.040
Lower lung field TB	136 (81.9)	28 (73.7)	108 (85.7)	0.084

The number and type of TB symptoms were similar across groups (*P* > 0.05), with cough (203/204; 99.5%) and sputum production (191/204; 93.6%) being the most common. Chest radiographs were available for 166 participants. Cavitary lesions were present in 107 (64.5%), infiltrates in 146 (88.0%), and lower lung field involvement in 136 (81.9%). Cavitary lesions were observed in 51.2% of TB–diabetes participants and 72.5% of TB-only participants (*P* = 0.014). Pulmonary infiltrates occurred in 79.5% vs. 92.0% of participants, respectively (*P* = 0.040). Lower lung field involvement was similar between groups (73.7% vs. 85.7%; *P* = 0.084) (Table 3). Sensitivity analysis showed largely consistent results. Cavitary lesions remained lower in TB-diabetes participants (51.2% vs. 68.5%; *P* = 0.015), while pulmonary infiltrates lost statistical significance (79.5% vs. 90.5%; *P* = 0.066). Lower lung field involvement remained similar (71.8% vs. 85.0%; *P* = 0.154) (Appendix Table A1). Among TB-diabetes participants with chest radiographs (39), cavitary lesions were observed in 38% (5/13) of previously diagnosed participants and 58% (15/26) of newly diagnosed participants.

### 3.4 Follow-up and TB treatment outcomes by baseline diabetes status

Among 139 participants completing all follow-up diabetes screenings, 104 were TB-only and 35 TB–diabetes at baseline. Eight TB-only participants (7.7%) had diabetes during follow-up, five of whom reverted to normal glucose by treatment completion. Of 20 participants newly diagnosed with diabetes at baseline, 13 had normal glucose by Month 3, and a further three by treatment completion. Two remained in the diabetes category throughout follow-up (Appendix Table A2). Among participants with previously diagnosed diabetes, poor glucose control persisted. The number with HbA1c > 9% increased from 8/15 at baseline to 11/15 at treatment completion.

Regarding anti-diabetic medication use, five of the 30 newly diagnosed participants (16.7%) started anti-diabetic therapy within 3 months, while those with previously diagnosed diabetes remained on treatment. Metformin was the most common drug (60% of prescriptions)—prescribed alone or in combination with other hypoglycaemics.

Month 2 smear results, available for 174 participants (132 TB-only, 42 TB-diabetes), were analyzed to assess early treatment response. Four TB diabetes participants (9.5%) and 18 (13.6%) TB-only participants remained smear-positive, a difference that was not statistically significant (*P* = 0.485). Follow-up sputum results were available for 120 participants at Month 5 and 127 participants at the end of treatment. Two participants remained smear-positive at Month 5, while no smear-positive results were recorded at the end of treatment (Appendix Table A3).

TB treatment outcomes were available for 193 participants. Overall, TB treatment adherence was high, with 80.8% (156/193) of participants maintaining uninterrupted daily dosing throughout the full treatment period. There was no significant difference between TB-diabetes and TB-only groups (*P* = 0.721). Most participants achieved favorable outcomes (175/193; 90.7%; cured = 125, treatment completed = 50), while 18 (9.3%) experienced unfavorable outcomes including two treatment failures, two deaths, and 14 LTFU). TB-diabetes participants had slightly fewer unfavorable outcomes than TB-only patients (6.8% vs. 10.1%; RR = 0.68, 95% CI 0.21–2.23; *P* = 0.515) (Table 4).

**Table 4:** TB treatment outcomes of study participants.

TB treatment outcome	Participants	TB-diabetes	TB-only	RR [95% CI]
	*N* (%)	*n* (%)	*n* (%)	
Favorable	175 (90.7)	41 (93.2)	134 (89.9)	1.04 [0.94–1.14]
Unfavorable	18 (9.3)	3 (6.8)	15 (10.1)	0.68 [0.21–2.23]

CI, confidence interval; RR, crude risk ratio.

## 4 Discussion

This study estimated the prevalence of diabetes among TB patients in Greater Accra Region and assessed its relationship with TB clinical presentation and treatment outcomes. Combined testing revealed many newly diagnosed diabetes cases at baseline. Previously diagnosed participants had persistent poor glucose control. Many participants who were newly diagnosed with diabetes at baseline had normal glucose by the end of TB treatment. Anti-diabetic medication use was recorded, with metformin most common. TB-diabetes participants had fewer cavitary lesions. Overall, TB treatment outcomes were similar between participants with and without diabetes.

The diabetes prevalence in our study (22.1%) exceeds previous reports among TB patients globally (15.3%) (8), across Africa (6.7%) (28), and in sub-Saharan Africa (9.0%) (9). In Ghana, earlier studies reported a prevalence of 9.4% (12), whereas the prevalence in our cohort was approximately 3.4 times higher than the estimated 6.5% in the general adult population (10). Comparable prevalence estimates have been reported in Nigeria (18.3%) (29), China (22.0%) (30), and India (22.6%) (31), suggesting consistency in some country-specific settings. Generally, estimates of diabetes prevalence among TB patients vary across settings (8), likely due to differences in screening methods and study populations (32). In particular, our use of self-report combined with FPG and HbA1c may have increased case detection. Ethnic differences in HbA1c-based diabetes diagnoses among TB patients have also been reported (33), potentially influencing prevalence estimates.

The high proportion of newly diagnosed diabetes cases highlights the potential for TB clinics to serve as strategic points for diabetes detection. The characteristics of participants with diabetes in our study align with expert recommendations for risk-based screening, which prioritize age ≥40 years, higher BMI, and a family history of diabetes (34). Although the highest odds of diabetes were observed among participants aged ≥60 years, we also noted a steady increase in odds with advancing age, supporting screening at younger ages.

Evidence on the association between diabetes and TB clinical presentation is mixed. In our cohort, TB-diabetes participants had fewer pulmonary cavities. This is consistent with a previous study, but contrasts with most reports, including the previous Ghana study (12), which reported higher cavitation. A few others found no difference (29, 35). Previous research shows that glycaemic control is more closely linked to radiographic severity than diabetes status alone, with poor control associated with more extensive cavitation (24). In our study, most TB–diabetes participants were newly diagnosed, which may have meant shorter diabetes duration. Also, although many previously diagnosed participants had poor glycaemic control they were receiving metformin. Metformin may reduce lung damage through immune modulation, enhanced autophagy, and inhibition of matrix metalloproteinases (36–39). Cavitation also varies by age and sex, with higher prevalence in males and lower prevalence in older patients (24). In our study, the TB–diabetes group had fewer males and were older on average, which may have contributed to the lower cavitation prevalence observed. Because pulmonary cavitation is a hallmark of PTB (40), its reduced frequency in patients with diabetes warrants caution in settings that rely heavily on clinical or radiographic features for diagnosis. Larger studies are needed to confirm our findings and better define the influence of diabetes severity, treatment, and patient characteristics on radiographic manifestations of pulmonary TB.

Contrary to some studies reporting an association between diabetes and unfavorable TB outcomes (3–5, 12, 41), we observed no significant differences in treatment outcomes by diabetes status. Month 2 sputum smear positivity, a known predictor of unfavorable outcomes (42), was also similar between groups and less frequent among TB-diabetes participants. Possible explanations for these findings include metformin use among some TB–diabetes participants and the high proportion of newly diagnosed cases. Metformin, beyond reducing lung damage, has been associated with reduced TB mortality (43) and improved treatment outcomes, including faster sputum conversion (44). In addition, it appears that many newly diagnosed participants had transient hyperglycaemia, with glucose normalizing with TB treatment. Transient hyperglycaemia has been associated with unfavorable TB outcomes in some settings (45, 46). However, this did not translate into unfavorable outcomes in our cohort. Furthermore, the lower prevalence of pulmonary cavitation among TB-diabetes participants may have further mitigated unfavorable outcomes. Cavitation has been shown to be a stronger predictor of unfavorable treatment outcomes than diabetes itself (47). Nevertheless, larger longitudinal studies are needed to confirm these findings and explain the underlying mechanisms.

This study has limitations. We did not use the oral glucose tolerance test, the diagnostic gold standard for diabetes, which may have resulted in missed cases. However, the combined use of FPG and HbA1c, both recommended for diabetes screening in TB patients (34), is expected to enhance diagnostic sensitivity. Mycobacterial clearance was assessed by sputum smear rather than culture, potentially underestimating persistent infection. Some participants missed scheduled glucose testing during TB treatment, limiting longitudinal data on glycaemic variability. Few participants with previously diagnosed diabetes constrained evaluation of glucose control in relation to TB outcomes. Limited diabetes management data precluded comprehensive assessment of treatment effects.

Despite these limitations, our findings support integrating diabetes screening into TB services in the Greater Accra Region to enable early detection and strengthen health system responses to the dual burden of communicable and non-communicable diseases. Further research is needed to identify effective strategies for integrating diabetes screening and care into routine TB services. In addition, larger studies in this setting incorporating comprehensive longitudinal monitoring of glycaemic variability, detailed diabetes management data, measures of diabetes severity, pharmacologic information, and relevant biomarkers are needed to clarify the impact of diabetes on TB presentation and treatment outcomes.

## Data Availability

The raw data supporting the conclusions of this article will be made available by the authors, without undue reservation.
