# Supplementary material for: Diabetes mellitus and its association with tuberculosis clinical presentation and treatment outcomes: results from a prospective cohort study in Ghana
Source: Front Public Health. 2026 Apr 9;14:1755479. doi: 10.3389/fpubh.2026.1755479 (PMC13102587; doi:10.3389/fpubh.2026.1755479)
Supplement: Supplementary file 1 [file Table_1.pdf]

Supplementary Material

Appendix Table A1: Sensitivity analysis of chest x-ray findings by baseline diabetes status

Radiological presentation	Cohort (*N* = 166)	TB-diabetes (*N* = 39)	TB-only (*N* = 127)	*P* value
	***n* (col%)**	***n* (col%)**	***n* (col%)**	
Cavitary lesions	107 (64.5)	20 (51.2)	87 (68.5)	0.015
Pulmonary infiltrates	146 (88.0)	31 (79.5)	115 (90.5)	0.066
Lower lung field TB	136 (81.9)	28 (71.8)	108 (85.0)	0.154

Appendix Table A2: Baseline and follow-up diabetes status of participants completing all three scheduled screenings during TB treatment

Baseline	Month 3	End of TB treatment	Total (*N* = 139)
No diabetes	No diabetes	No diabetes	95
No diabetes	No diabetes	Diabetes	3
No diabetes	Diabetes	No diabetes	5
No diabetes	Diabetes	Diabetes	1
Diabetes	No diabetes	No diabetes	13
Diabetes	Diabetes	No diabetes	3
Diabetes	No diabetes	Diabetes	2
Diabetes	Diabetes	Diabetes	17

Appendix Table A3: Sputum smear results of participants at Months 2, 5, and end of treatment by baseline diabetes status

Smear status	All	TB-only	TB-diabetes	*P* value
	n **(col%)**	***n*** **(col%)**	***n*** **(col%)**	
**Month 2**	***N*** **= 174**			0.485
Negative	152 (87.4)	114 (86.4)	38 (90.5)	
Positive	22 (12.6)	18 (13.6)	4 (9.5)	
**Month 5**	***N*** **= 120**			0.452
Negative	118 (98.3)	88 (98.9)	30 (96.8)	
Positive	2 (1.7)	1 (1.1)	1 (3.2)	
**End of treatment**	***N*** **= 127**			-
Negative	127 (100.0)	99 (100)	28 (100)	
Positive	0	0	0	
